# Perceptions, attitudes and challenges about obesity and adopting a healthy lifestyle among health workers in Pietermaritzburg, KwaZulu-Natal province

**DOI:** 10.4102/phcfm.v9i1.1276

**Published:** 2017-06-22

**Authors:** Patrick Simfukwe, Brian Van Wyk, Charl Swart

**Affiliations:** 1 School of Public Health, Faculty of Community and Health Sciences, University of Western Cape, South Africa

## Abstract

**Background:**

The prevalence of obesity is reported to be high and increasing among health workers, in both high-income and low-income countries, which in turn is a common risk factor for all non-communicable diseases. This is alarming, as health workers not only serve the community’s health needs but should also serve as role models for a healthy lifestyle. It is therefore important that obesity among health workers is addressed and prevented.

**Objective:**

The aim of the study was to explore perceptions and attitudes about obesity among health workers in three selected hospitals in Pietermaritzburg, and to identify the barriers to adopting a healthy lifestyle.

**Methods:**

An explorative and descriptive qualitative study design was used, utilising in-depth interviews for data collection. A total of 18 health workers from the three selected hospitals in the metropolitan were individually interviewed. Thematic analysis was done, using a priori themes that were derived from the Health Belief Model.

**Results:**

All health workers were aware of the negative consequences of being overweight or obese. However, only a few of the participants chose to adopt a healthy lifestyle. Barriers to adopting a healthy lifestyle included institutional as well as attitudinal factors.

**Conclusion:**

Public healthcare facilities need to invest in their work force by giving health workers access to physical exercise facilities and affordable healthy food within the hospital. Health organisations should introduce health behaviour change programmes to combat negative established cultural norms among health staff.

## Introduction

Overweight and obesity are serious public health problems. The prevalence of obesity in the United States of America (USA) in 2004 was reported to be 27% and 32% among men and women, respectively.^1^ In the slum residents in Nairobi, 43.4% of women and 17.3% of men were reported to be overweight or obese in 2008–2009.^2^ In a nationally representative sample in South Africa, it was found that 29% of men and 56% of women were overweight or obese in 2013.^3^ Recent studies suggest that the prevalence of obesity among health workers is a growing global phenomenon.^4^ This is alarming, as health workers not only serve the community’s health needs but should also serve as role models for a healthy lifestyle.

The high prevalence of obesity in various countries includes health workers. Abbate, Giorgianni, Munao, Beninato, D’Arrigo, D’Arrigo and Brecciaroli reported that 13.3% and 13.6% of male and female health workers, respectively, at a large hospital in Messina, Italy, were obese.^5^ Al-Haddad, Al-Haddad and Al-Sayyad also reported a high prevalence (70%) of obesity among health workers at Salmaniya Medical Complex in Bahrain between 2009 and 2012.^6^

Studies in middle-income countries showed similar results. The study conducted in 2013 in India reported that 37.5% of nursing students were overweight or obese,^7^ and these results were supported by Selvaraj and Sivaprakasam who found that 24% of the medical students at a medical college and research institute in south India were overweight, and 8.6% were obese in 2012.^8^

Several African studies have reported similar findings. In Ghana, the prevalence rate of overweight or obesity among health workers in 2013 was 42.4% for women and 32.9% for men,^9^ while in Nigeria 27.3% of the health service providers were obese and 44.7% were overweight in a facility-based study in 2013.^10^ This was supported by Garrido, Semeraro, Temesgen and Simi, who reported that between 2007 and 2008, 28.7% of the health workers in a privately funded hospital in Botswana were obese while 27.3% were overweight.^11^

Research in South Africa found that many health workers are overweight or obese. Onyebukwau reported that 29.7% and 41% of the health workers at the Mafikeng Provincial Hospital were overweight and obese, respectively.^12^ Another study, conducted at a public tertiary hospital in Pretoria in 2008, found that 75% of health workers were obese.^13^ These results were further supported by another study among nursing students in the Eastern Cape province, which reported 49.7% of students were obese in 2007.^14^

These studies illustrate that weight problems and obesity among health workers is a serious public health concern in South Africa. However, little is known about the perceptions about obesity and the attitudes of health workers towards obesity. It is therefore necessary to explore the perceptions of obesity among this important group in public health. It is assumed that health workers should be knowledgeable and serve as role models in tackling this public health problem. This study explored health workers’ perceptions and attitudes about obesity in Pietermaritzburg, KwaZulu-Natal (KZN) province.

## Methods

### Study design

An explorative and descriptive qualitative study was conducted to investigate the perceptions and attitudes of health workers about obesity in Pietermaritzburg Hospital complex, as health workers participating in the study were able to share their experiences with regard to obesity, as well as the barriers encountered to following a healthy lifestyle. Participants were interviewed in their respective hospitals where they are employed.

### Description of study settings

The study was conducted at Edendale, Northdale and Grey’s Hospitals in uMgungundlovu district in Pietermaritzburg. Edendale Hospital historically served the african population, Northdale the indian population and Grey’s the white population. The district covers an area of 125.2 km^2^ and has a population of 223 448.^15^ Its population comprises 70% african, 8.4% indian/asian, 14.2% white and 6.9% mixed race.^15^

### Classification of body weight using body mass index

Participants were asked to write their height and body weight on the consent form upon accepting that they are willing to participate in the study. Using anthropometric measurements, total body fat was estimated by the body mass index (BMI), which was calculated as body weight in kilograms divided by height, squared, in metres. Participants were classified according to their BMI as shown below:

Normal: 18.5–24.9

Overweight: 25.0–29.9

Obese: > 30

### Study population and sampling

The study population was constituted of health workers from the mentioned hospitals who met inclusion criteria. Six health workers from each hospital site were recruited purposively in the study sample. Physiotherapy managers informed various component managers about the proposed study, who then assisted in recruiting suitable participants from their respective components. The researcher purposively selected two obese health workers from each institution into the study sample, which allowed these individuals to share perceptions about obesity. All participants were recruited using purposeful sampling. The component managers in different clinical areas were requested to recruit four staff members from their component to ensure representation from as many of the professional categories as possible.

### Development of in-depth interview guides

Constructs from the Health Belief Model (HBM) were used to guide development of questions for two interview guides. Questions were based on the following constructs: perceived susceptibility to non-communicable diseases (NCDs), perceived seriousness of obesity, perceived benefits of leading a healthy lifestyle, perceived barriers to leading a healthy lifestyle, cues to action and self-efficacy.^16^

The interview guide was tested by interviewing a nursing college lecturer, who specialised in qualitative research, to establish if the questions were yielding answers that were appropriate to the study topic.

### Data collection

In-depth, one-on-one interviews were conducted to collect data, as the researcher wanted to be able to capture verbal and non-verbal communication, as well as make participants comfortable to be able to fully engage in the discussion topic during the interview.^17^

Two different semi-structured interview guides were used to guide the in-depth interview for obese/overweight and normal weight health workers, respectively. This was done in order for the researcher to get perceptions on weight issues from both persons who are obese/overweight and those with normal weight because their challenges and attainments may differ. Participants were stratified according to self-reported weight type (i.e. normal body weight vs. obese). All the interviews were conducted in English and were audio recorded using an electronic voice-recorder. Non-verbal communication was recorded in the field notes that the researcher took during the interviews.

### Data analysis

The analysis of the data started with the researcher performing the interview listening carefully during the discussions and when transcribing the interviews. Tape-recorded data were transcribed verbatim and analysed manually. Thematic content analysis was done to realise the objectives of the study.

The HBM was used to inform the analysis.^16^ For this purpose, the following six predetermined themes were applied to the data: perceived susceptibility to NCD, perceived severity of obesity, perceived benefits of leading a healthy lifestyle, perceived barriers to leading a healthy lifestyle, cues to action and self-efficacy.^16^ These themes were determined before the individual interviews to purposefully guide the researcher, as they relate closely with health behaviour at the individual decision-making level. The data were then rechecked by the researcher and the research assistant for consistency and dependability to ascertain that no major categories were omitted.

### Ethical consideration

Ethics approval for the study was granted by the Senate Research committee of the University of the Western Cape: registration No: 14/10/40, KwaZulu-Natal Department of Health Research and Knowledge Management [(KZN DOH – HRKM) REF: KZ 2015RP 920], and the three participating hospitals.

Participants were also assured that they could choose to stop participating at any time without giving an explanation, should they desire to do so. Those who wished to seek medical help were assisted in doing so by the researchers referring them to the appropriate service provider.

## Results

The study results were first reviewed by describing the sociodemographic characteristics of the participants in Table 1 below. Eighteen participants were interviewed. The study sample consisted of 14 women and 4 men, and the majority were between 46 and 50 years old. There were seven African participants, six Indian participants and five White participants in the sample. There were more African female participants, which is reflective of the health worker population in Pietermaritzburg medical complex. In the complex, the White population is proportionally the lowest, and the number of White health workers is thus correspondingly lower. Ten participants self-identified as having normal weight, two as overweight and six as obese. The three hospitals were chosen according to their predominant racial group in their staff constitution, namely African, Indian and White.

**TABLE 1:** Sociodemographic characteristics of study participants.

Characteristics	Frequency
**Gender**	
Male	4
Female	14
**Age (in years)**	
< 40	2
41–50	13
> 51	3
**Ethnicity**	
African	7
Indian	6
White	5
**Self-perceived body weight**	
Normal	10
Obese	6
Overweight	2

### Predetermined themes

The six predetermined themes are indicated in Table 2.

**TABLE 2:** Classification of main themes, sub-themes and codes.

Themes	Sub-themes	Codes
Perceived susceptibility to NCDs	Perceived susceptibility to NCDs	Eating habits Exercising is uncomfortable Dependence on machinery
Perceived severity of obesity	Being overweight/obese limits work efficiency	You cannot get close enough to patients You cannot manipulate patients effectively
	Consequences of obesity	Higher risk of NCDs Heart diseases It can kill you Poor quality of life
Perceived benefits of leading a healthy lifestyle	Productivity of workforce	Healthy workers
	Prevention of NCDs	Taking care of ourselves first Health population
	Physical appearance	Enhanced beauty Enhanced body image
	Role modelling	An example to clients and the community
Perceived barriers to leading a healthy lifestyle	Beliefs about obesity	Thin people are sick/unhappy Fat people are happy and healthy
	Utilisation of health promotion services	Lack of reliable information on diet Lack of accessibility to healthy food Lack of physical exercising facilities Lack of management support Lack of time Poor salaries
	Cultural norms	Big is beautiful
Cues to action	Personnel diagnosed with health problems	Workmates suffering from NCDs Family members suffering from NCDs
	Appearance	Limited wardrobe Clothes not fitting properly
	Posters	Display of posters on NCDs
Self-efficacy	Goal setting	Starting an exercise programme Progress in programme adherence
	Self-motivation	Coping with the new programme New eating habits Weight reduction Losing weight is motivating
	Social support	Group exercises Family members help Eating buddies

NCDs, non-communicable diseases.

### Perceived susceptibility to non-communicable diseases

All the participants acknowledged that they were aware that their choices of food consumption contribute to obesity and that people can eventually die from obesity-related conditions.

### Perceived seriousness of obesity

#### Obesity is limiting

Certain obese health workers find it difficult to interact physically with their clients, as their size limits their mobility and their ability to handle patients. This experience was shared by a radiographer who said that he experiences difficulties in performing procedures on patients:

‘It is absolutely not healthy, because the overweight can limit the productivity of a health workers. They will be more vulnerable to injury, because with the movement you know there will be limitations to move easy and flexibly.’ (Radiographer, Male, Overweight)

#### Consequences of obesity

Participating health workers were aware of the risk factors of obesity and they reported that obesity has severe consequences on the individual’s performance at work and that quality of life is compromised.

‘Haa! Because of overweight the doctors say that I have hypertension and I am prone to have cardiac failure and diabetes. But I have not got them yet. Haa. The only thing I am having now, is hypertension and this makes it difficult for me to cope at work.’ (Nurse, Female, Obese)

‘Obesity is not good. It will kill you by decreasing your life expectancy. I was nearly boarded out of work by department of health because of severe backache. I was on long extended sick leave. Imagine what could happen to my family.’ (Nurse, Female, Overweight)

### Perceived benefits of leading a healthy lifestyle

#### Productivity of workforce

Participating health workers who follow a healthy lifestyle felt that they were more effective and productive at work. Participants referred to health workers who do not exercise as lazy and slow to attend to patients.

‘I think there is a significant increase in the number of health workers that are obese/overweight and I have noted it specifically in the wards; like obese health workers are unable to move patients as quickly as they should because they do not have strength to lift or move patients, especially the nurses. They can also not move close enough to lift the patient properly and they struggle walking up the stairs so they always wait for lifts.’ (Nurse, Female, Normal bodyweight)

The study participants also reported that it was vital to keep the work force healthy as this was beneficial to the communities they serve. They reported that it was difficult to control staff absenteeism because of sickness.

‘Health workers are the engine of the hospital, because without staff you cannot function; so taking care of your staff is the right thing to do. To keep your staff protected and healthy is important, because no system can make staff healthy apart from healthy education. This will improve their health and productivity. Systems like incapacity leave and others on absenteeism may not help you.’ (Doctor, Male, Normal bodyweight)

#### Prevention of non-communicable diseases

The study participants expressed the perception that health workers who keep fit and follow proper diet were more likely not to suffer from NCDs. Proper diet was referred to as eating a healthy diet, which consisted of food that was not rich in saturated fats and oil, and included a combination of five fruits and vegetables per day.

‘It’s beneficial to start exercising on a small scale with realistic goals, and also to give up sugar. Myself, I do exercise 4-5 times a week for at least an hour. You can see how effective I am as a person.’ (Nurse, Female, Normal bodyweight)

Participating health workers were aware that NCDs are preventable by following a healthy lifestyle of eating healthy food and regularly doing physical exercise.

‘Like I said before, there are a lot of conditions associated with obesity. We need to maintain a healthy life style and remain more active. We need to exercise regularly in order to remain healthy, even if it means just walking around the neighbourhood.’ (Nurse, Female, Normal bodyweight)

#### Physical appearance

The study participants reported that health workers following a healthy lifestyle were in a better state of health, appeared healthier and had enhanced self-image. This, according to the participants, could be seen in the way they conducted themselves at work and when they were in public.

‘I feel that health workers who start following a healthy lifestyle to be healthy, but they get even more encouraged when the adopted lifestyle makes them look beautiful.’ (Nurse, Female, Normal bodyweight)

#### Role modelling

The researcher also reported that clients and patients find it easy to take the advice received from health workers who look physically healthy and this gives them motivation to look like these health workers. They assume that health workers who look healthy know what they are doing and as such they are more willing to be advised by them.

‘I think it will be a good thing if the health worker is an example. Imagine that when patients came to the hospital and see that the health workers are not healthy, it becomes very difficult for them to trust what the health worker is telling them.’ (Doctor, Male, Normal bodyweight)

### Perceived barriers to leading a healthy lifestyle

Despite having knowledge about obesity, some participants conceded that they choose to eat unhealthy food as it is convenient and there is limited availability of healthy food options in the hospital.

‘I am not buying from the cafeteria. I bring my lunch from home because I am following orders from the dietician. There they sell oily food. Although there are some salads, I do not still buy in the cafeteria. Some staff eat fat cakes mixed with polony.’ (Nurse, Female, Obese)

‘I know here in the cafeteria, the most food sold is oily curry and rice at times with salads but no fruits. Anyway, at times I have no option but to buy and eat.’ (Nurse, Female, Overweight)

Participants had different views on their reasoning behind their choice of unhealthy food: a professional nurse narrated that she eats any food she wishes to eat without taking into account the health value of the food. Certain health workers continue to indulge in unhealthy food despite their knowledge of the consequences. There are no clear reasons as to why, although some mentioned simply giving into cravings for these foods.

‘Yaaa. These junk food we like eating and not abiding to lifestyle modification. We do not care; we just eat whatever you find. You just feel like eating that time – no matter what. You know, most of the time we just like these junk foods, because it is nice and accessible. To roll (spoil) yourself you, just go there in the restaurant and buy McDonald’s triple or double burgers with chips.’ (Nurse, Female, Obese)

Participating health workers reported that their unhealthy behaviour is influenced by the expectations within their respective communities that when a person is doing well financially, it has to be reflected by consumption of takeaway food from fast food outlets such as McDonalds and Kentucky Fried Chicken (KFC), and serving large portions of food during meal times at home. This was cited by an obese social worker:

‘In our family, food is not measured and visitors eat as much as they can so that they will go back to their homes full.’ (Nurse, Female, Obese)

The study also found that certain participating health workers do not like exercising or controlling their diet, as it is difficult and laborious:

‘Exercising is not one of my strongest points; I would rather drink slimming tablets, because exercising is like punishment.’ (Nurse, Female, Obese)

Some participants reported that the makes they feel lazy to go exercise because they get physically tired at the end of each shift.

‘Exercising is good, but I operate an x-ray machine for 8 hours and the only time I have to relax, is watching news and soapies at home.’ (Radiographer, Male, Obese)

The above views were complemented by the dental technician who reported that he found it difficult to exercise after work but preferred to often watch television instead. The habit of inactivity predisposes a person to obesity, yet certain people are still under the impression that this habitual behaviour cannot lead them to becoming obese:

‘After work, I watch television from 17:00 until I go to sleep at about 22:00 every day. I think this is in the family.’ (Nurse, Female, Normal bodyweight)

#### Beliefs

Some participating african health workers believed that people who are healthy are not supposed to be skinny. This belief was prevalent among some of the african participants who associated being skinny with poverty and ill health. Furthermore, this sentiment was echoed by some of the respondents who have had ill relatives or friends. Some health workers reported being discouraged from adopting a healthy lifestyle for fear of being associated with having HIV or AIDS.

‘Some people, when they see a thin health worker, they start considering that person to be sick with HIV/AIDS and they feel uncomfortable to be attended to by her.’ (Radiographer, Male, Obese)

The other belief, which was found to discourage health workers from adopting a healthy lifestyle, was that some of the african males and females interviewed associated overweight or obesity with good living, progress and prosperity. They believed that when people have enough food, they should eat as much as possible as this was a reflection that they had enough money to spend on food and, as such, they were considered to be doing well.

‘As Africans, we believe that the family and friends should eat as much as possible, so food portions are not measured because people’s appetite on food is different.’ (Nurse, Female, Overweight)

#### Utilisation of health-promoting services

The study found that participating health workers are overworked. Some workers, such as nurses, worked from 06:45 to 18:45 during normal working days and on weekends. Junior medical officers worked from 07:30 to 13:00 the following day – a total of 29 hours at a time. The period health workers were on duty was long and at the end of the shift they were exhausted, and as such it was difficult to do physical exercise while at the hospital or go to the gymnasium outside the institution (after hours).

‘A junior doctor will come for work at 07:30 hours in the morning; then they leave the next day at midday, so they are working for 29 hours. They are exhausted; so to go and exercise will be very difficult. This could be the main reason why health workers do not exercise.’ (Nurse, Female, Normal bodyweight)

It was further found that the long working hours were perceived to impact negatively the health workers’ ability to prepare healthy meals at home. They arrived home late and tired, so they usually bought takeaways from fast food outlets, like McDonalds and KFC, which were still open.

‘Spending much time at work makes it difficult for health workers to prepare home meals because they are too exhausted to cook and as such they end up buying any food available, like junk food.’ (Doctor, Male, Normal bodyweight)

The participating hospitals utilised the space in their physiotherapy departments for staff to exercise. However, this was not ideal because the available times (for exercise) were limited to 12:00 to 16:00, and this did not give everyone an opportunity to utilise the exercise facilities.

‘We come in at 08:00 hours and we knock off at 16:00 hours and by the time we try to get to the facility, people who are running the facility, also knock off, so it really difficult to access it. Maybe dedicating some of the staff members to take over from those who knock off at 16:00 hours, and carry over until 18:00 hours.’ (Nurse, Male, Overweight)

Participating health workers also reported that poor salaries were another barrier to adopting a healthy lifestyle. Poor remuneration made it difficult to afford healthy food: even if they desired to purchase pre-packed health foods or just fruit and vegetables, it was too expensive.

‘I know here in the cafeteria, the most sold is curry and rice, at times with salads, but no fruits. Anyway, they do have a tuck shop where they sell fruits, but they are slightly expensive; like one banana is R3.’ (Nurse, Female, Normal bodyweight)

The study also reported that hospital management was not very supportive of the health needs of staff. They offer very limited motivation in supporting staff and maintaining the physiotherapy departments which are usually utilised for health promotion. For example, nurses had meal times at very close intervals. The intervals in between meals were too short for proper digestion to take place.

‘Like our teas and lunches are too close, if you had breakfast at 09:00 hours to 09:30 hours and your lunch is 12:00 hours, in that gap you are still full, but because you are short staffed, we do not have a break like at 15:00 hours, so we are forced to eat your lunch at 12:00 hours, even if you are still full. But by 15:00 hours you are hungry, but there is no tea time break. I hope the management can do something to improve our eating pattern.’ (Nurse, Female, Obese)

There was a policy from the KwaZulu-Natal department of health which allowed health workers to exercise for 2 hours while on duty. Some participants reported that despite the policy, it was difficult to find time because of staff shortages.

‘In a ward of 45 beds, at times there are only five nurses covering all the ward and as such it is difficult to find time to go exercise in the afternoons when the physiotherapy department allows us to do so.’ (Nurse, Female, Obese)

#### Cultural norms

Participating white and indian health workers preferred slim bodies, as they associated a slim body with being healthy and beautiful. However, participating african health workers preferred body sizes which were either overweight or obese, because these body types were associated with beauty and success.

‘In my culture, we believe that big woman is the one. Comparing our culture with Indian culture; they like thin women, but us we like big women because the big ones are healthy.’ (Radiographer, Male, Overweight)

These ideas were also shared by other African health workers including females who said that a women has to be thick and should have big hips.

‘If you are thick, you are considered to be attractive to men, but if you are skinny, you are considered not. I know people want to please other people, but it also depends on yourself; like me, I want to be between overweight and obese.’ (Nurse, Female, Normal bodyweight)

### Cues to action

#### Personnel diagnosed with health problems

Participating health workers who had colleagues or family members diagnosed with NCDs were motivated to seek medical advice and to modify their lifestyles. Some of them were motivated to attend disease screening at clinics like the diabetic clinics, to change their unhealthy eating habits or to incorporate daily exercise in their lives.

‘I think, the main reason for honouring appointments is the fear of suffering from NCDs and prevention of disease progression. It may not suit all health workers to be assisted in clinics, but it might suit some.’ (Doctor, Male, Normal bodyweight)

‘Basically, I gained a lot of weight with incorrect lifestyle; so I went to see a dietician about two months ago.’ (Nurse, Female, Obese)

#### Appearance

Participating health workers were conscious about their body image, both at work and in public places. They were worried when their clothes or uniforms started to fit tightly, because it would be uncomfortable and meant that they need to change their wardrobe. The fear of not looking good in public and buying new clothes motivated some health workers to change their lifestyle so that they can maintain a normal weight.

‘Ladies need to look good even at work, whether in uniform or not, to boost our own ego, but changing a wardrobe is expensive.’ (Nurse, Female, Normal)

#### Posters

The study participants further reported that the ongoing awareness campaign in the institutions was a motivation to some health workers to change their lifestyles. There are posters on the notice boards in some hospitals on complications of obesity and other diseases, and these were reported reminders to health workers to start looking after their health.

‘The message on posters made me start running or at times just walking with my dogs from my home to the shopping centre, and that takes me about an hour.’ (Nurse, Female, Normal bodyweight)

### Self-efficacy

#### Goal setting

Some participating health workers set goals for themselves to reach in order to improve their self-efficacy. They set goals to attain in their exercising and eating programmes. One health worker mentioned that she used to exercise twice a week, but that she has increased the frequency to three times. Her confidence increased after noticing her own progress:

‘I have noted a progress in my exercising programme so now, I wish to exercise five times a week.’ (Nurse, Female, Obese)

#### Self-motivation

Participating health workers reported that their self-efficacy improved when they noted improvements in their weight control programmes. Someone narrated that he was finding it very difficult to control his craving for fried meat. After starting a controlled diet programme of eating cooked meat, vegetables and fruits, he noted a reduction in his weight, and this has motivated him to continue and has improved his self-efficacy.

‘I was weighing 120 kg, now I am 100 kg just after one year of starting my weight control programme. This makes me feel good.’ (Radiographer, Male, Obese)

#### Social assistance seeking

Participating health workers felt that they needed someone to encourage them to continue exercising or to control their diets. A need was expressed for an exercise partner, in order to stay motivated. This could be a spouse, friend or family member.

‘I think the best is to get a partner. If they have got kids, you should invite them as gym partners; that is what I do myself. I go for running on the road with my grandson. This is very helpful, because in most cases, he encourages me to go run even if I might be feeling lazy that day.’ (Nurse, Male, Overweight)

The study also found that participating health workers find confidence when they have someone to share healthy meals with during meal times. The eating buddy helps them to adhere to good eating habits, thus boosting their self-efficacy.

‘We share the meals we prepared from home while at work during our meals times and this helps to monitor our eating habits. This does assist especially that we even share the recipe.’ (Nurse, Female, Obese)

## Discussion

This study found that South African health workers in Pietermaritzburg medical metropolitan complex were aware of the negative consequences of being overweight and obesity. As such, some health workers had already realised that the chances of their suffering from these conditions were high and they were now involving themselves in health-promoting programmes such as modifying their eating habits and doing physical exercise. The results of the current study are consistent with studies that were done in the USA.^18,19^ Kim, Harrison and Kagawa-Singer reported that Americans were aware of the consequences of an unhealthy lifestyle but because of lack of time, money and adequate space for doing physical activities, regular exercise was a challenge.^18^ These findings are supported by Agne et al.^19^ who concluded that their study participants expressed awareness of the obesity epidemic and desire to lose weight, but it was difficult to exercise because of similar challenges as mentioned by Kim et al.^18^

Participating health workers with colleagues or family members suffering from NCDs were motivated to seek medical advice and to modify their lifestyle. Some of them needed assistance from family and colleagues to achieve their goals. This is consistent with Dalais’ findings who concluded that some participating educators expressed that they wanted support from family members or friends to comply with a weight loss programme.^20^ Studies conducted in South Africa also show the same trend. Puoane, Fourie, Shapiro, Rosling, Tshaka and Oelofse found that more than half of community health care workers in the Eastern Cape province tried to lose weight by reducing food consumption or taking slimming tablets in order to reduce the chances of suffering from NCDs.^21^ These findings are supported by other South African studies.^13,20,21^

Hospitals in the current study have a limited selection of healthy food and, as a result, health workers buy the (unhealthy) food which is available in the institution. The researcher observed that food outlets within the hospitals sell beef and chicken curries without fruit or vegetables. Fruit is mainly sold by vendors outside the hospitals but is expensive. This finding regarding the high cost of healthy food is supported by Phiri, Draper, Lambert and Kolbe-Alexander who found that the high cost of healthy food in hospital cafeterias was a barrier to nurses buying healthy food at work and from consuming the required daily quantity of fruit and vegetables.^22^ A few participating health workers reported bringing cooked food from home to avoid buying unhealthy food at work. Home-cooked meals are the best, but some participating health workers mentioned that because of long working hours it is difficult to prepare home meals. These findings are supported by Dalais who reported that educators feel frustrated because of their heavy workloads and lack of time available for personal use.^20^

In this study, certain obese health workers were finding it difficult to interact physically with their clients, as their size limits their mobility and their ability to handle patients. This was in agreement with Agne who reported that Latin Americans associated weight gain with the presence of multiple physical symptoms of obesity, discomfort and reduced physical capability, such as breathlessness, fatigue and low energy.^19^ This was further supported by Dalais in the study on educators.^20^

Participating health workers alluded that obesity has severe consequences on individual performance at work due to depression or stress. According to Phiri et al., a person who is sickly fails to work effectively and efficiently, and this impacts negatively other staff members.^22^ The study also reported that a disadvantage of being obese was having to buy a new wardrobe when clothes get small or tight fitting. This is supported by Dalais who reported that this could act as a motivation for losing weight, as was found among a group of educators.^20^

This study’s participants further reported that health workers who led a healthy lifestyle were observed to be more productive and performing their work more effectively. These results are similar to the findings from previous studies in South Africa.^20,21,22^

In the study, participating health workers who kept fit and followed a healthy diet had enhanced self-image. This is consistent with Agne’s findings that Latin Americans considered weight loss as a way to improve personal health and well-being.^19^ Some local studies support these findings; Mvo, Dick and Steyn reported that some women in their study gave practical reasons why they are not comfortable when they are big citing that they are uncomfortably hot or heavy and they are unable to wear clothes that others gave them, but no health reasons were given.^23^ This was supported by Puoane, Tsolekile and Steyn in their study where some of the girls cited that despite associating fatness with dignity, they also linked it with the risk to suffer from diabetes and high blood pressure.^24^ This study equally reported that clients and patients like to be attended by health workers who are role models in the way they present themselves. This is consistent with Oberg and Frank^25^ and Frank et al.^26^ who reported that patients emulate the behaviour of their physicians.

Nevertheless, participating african health workers believed that people who are healthy are not supposed to be skinny. Many other studies have confirmed this cultural perception that a thin person is suspected of illnesses such as TB and HIV or AIDS. Mvo et al. reported that some of their study participants viewed weight gain not to be associated with any negative connotation but felt to be attractive.^23^ Puoane et al. supported these findings because some participating girls believed that thinness is associated with illness and poverty.^24^ In the current study, participants believed that, when people have enough food, they should eat as much as possible as this is a reflection that they have enough money. This belief discourages health workers from adopting a healthy lifestyle. This is consistent with local South African studies.^23,24^

Physiotherapy departments, which are being used for physical activities, are primarily there to serve patients; thus, these facilities are only open to staff at times when there are few patients. This limits access to the facility. This is consistent with local studies. Skaal and Pengpid^13^ reported that some of the participants in their study had no time to exercise because they are too busy at work, and Dalais^20^ reported the same reason in their study on educators. In addition, some health workers find it difficult to get time for physical activities or to consult dieticians because of heavy patient workloads and staff shortages in the hospitals.^21^

## Conclusions

Despite health workers having knowledge of obesity, they cannot act without institutional support. Workplace health promotion programmes focusing on increasing opportunities for physical activities and availability of healthy food choices should be established to encourage long-term well-being of a health worker. The hospitals need to enable health workers to access physical exercise facilities and provide affordable healthy food options. Physical activities facilities should be made available so that they fit more conveniently with health workers’ work schedule, so that those who want to make use of it can do so.

Healthy food should also be made accessible and affordable by the hospital management, facilitating discussions and agreements between hospital cafeteria operators and food suppliers so that healthy food can be made available at affordable prices. Tailored health behaviour promotion programmes on obesity and other NCDs should be implemented in health institutions, to counter cultural norms which advocate overweight body size as desirable.

As an important limitation to the study, it needs to be recognised that the results of this study cannot be generalised to all health workers in South Africa because of the small sample size and the particular study settings that were chosen.
